# The Validity and Reliability between Automated Oscillometric Measurement of Ankle-Brachial Index and Standard Measurement by Eco-Doppler in Diabetic Patients with or without Diabetic Foot

**DOI:** 10.1155/2017/2383651

**Published:** 2017-05-09

**Authors:** Jing Ma, Min Liu, Dawei Chen, Chun Wang, Guanjian Liu, Xingwu Ran

**Affiliations:** ^1^Diabetic Foot Care Center, Department of Endocrinology and Metabolism, West China Hospital, Sichuan University, Chengdu 610041, China; ^2^Chinese Cochrane Center, Chinese EBM Center, West China Hospital, Sichuan University, Chengdu 610041, China

## Abstract

**Objective:**

To evaluate the concordance between oscillometric ABI and standard Doppler ABI in diabetic Chinese patients with or without diabetic foot.

**Methods:**

230 consecutive diabetic patients (*n* = 459 limbs) were included. The right and left ABIs were determined with both devices by the same investigator. The concordance and agreement were assessed by kappa index and the Bland-Altman method.

**Results:**

The average Doppler ABI was 1.003 ± 0.286 on the right and 0.990 ± 0.287 on the left, while oscillometric ABI was 1.002 ± 0.332 and 0.993 ± 0.319, which had no significance. The average time for oscillometric ABI was 8.600 versus 16.980 minutes for Doppler ABI (*p* < 0.001). There was good agreement between the two measurements, with a kappa value of 0.869 on the right and 0.919 on the left. Regarding the Doppler ABI as the gold standard, the accuracy, sensitivity, specificity, +LR, and −LR of oscillometric ABI reached 95.22%, 94.34%, 95.48%, 20.873%, and 0.059% on the right. For the left, it was 96.94%, 96.43%, 97.11%, 33.364%, and 0.036%.

**Conclusions:**

The oscillometric measurement is a reliable, convenient, and less time-consuming alternative to standard Doppler ABI in patients. It should be widely used for PAD detection.

## 1. Introduction

Peripheral arterial disease (PAD) is a common manifestation of atherosclerosis in patients with diabetes in China [1]. The prevalence of PAD in patients with type 2 diabetes aged 50 years and older is 19.47%~23.8% [2, 3]. PAD is significantly associated with an increased risk of cardiovascular morbidity and mortality [4, 5] and independently related with impaired lower extremity functioning [6]. Further, PAD patients with diabetes had a significantly increased risk for death within 10 years than did the PAD patients without diabetes [7]. However, most of PADs are asymptomatic; evidence suggests that there is currently a lack of awareness regarding PAD among physicians and patients, leading to underdiagnosis and undertreatment [8]. To reduce the cardiovascular morbidity and mortality, it is very important for early screening, diagnosis, and treatment of PAD. Measurement of the ankle-brachial index (ABI) is a simple and noninvasive method and generally recommended for middle-aged populations with elevated cardiovascular risk levels for the screening and diagnosing for PAD [9]. Eco-Doppler is considered the gold standard for ABI test; the cut-off values for PAD are 0.9 and 1.4 [10]. However, it requires a trained technician, it is time consuming, and it has a high intraobserver variability of 10%, which limited its routine use in clinical practice and did not seem suitable for the screening of PAD in primary care consultations [11].

To overcome such obstacles and simplify measurement procedures, automatic devices have been developed. Some recent studies have shown that the automated oscillometric method appears to be convenient and useful compared with eco-Doppler [12, 13]. The oscillometric method can be suitable for screening of PAD in the community due to having a simple procedure, being easy to perform, and not requiring training [14–17]. But some studies showed that it cannot be recommended as a reliable method for ABI measurement and few subjects had low ABIs [18–21]. Thus, we aimed to evaluate the concordance between automated oscillometric measurement of ABI and the standard measurement by eco-Doppler in diabetic Chinese patients with or without diabetic foot.

## 2. Methods and Patients

### 2.1. Study Population

**Figure 1:**
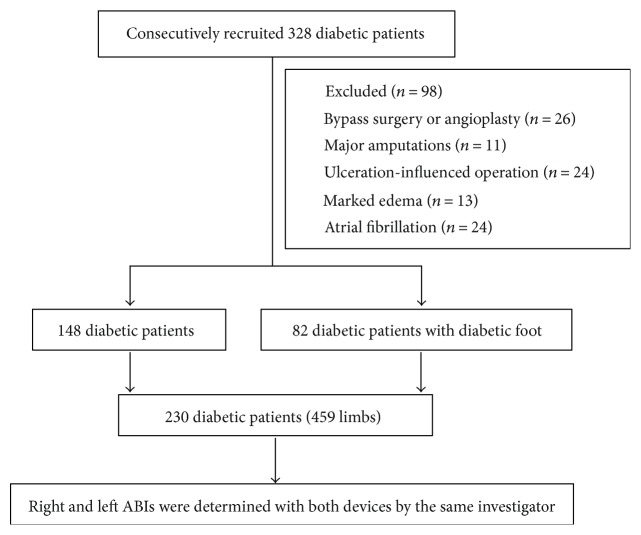
Flow chart of the study.

328 consecutive patients with diabetes who were admitted to the Diabetic Foot Care Center, Department of Endocrinology and Metabolism, West China Hospital, from May 2013 to June 2014 were recruited. Participants were excluded if they reported any history of previous bypass surgery or angioplasty, any major amputations on the lower or upper limbs, marked edema of one or both feet, and atrial fibrillation. Finally, 230 consecutive patients with diabetes (*n* = 459 limbs, mean age 61.28 ± 14.50 years, 126 men, 82 diabetic foot) were included our study.  Figure 1 presented the flow chart of the study. The study protocol, patients' informed consent forms, and other study related documents were reviewed and approved by the ethics committee of the West China Hospital, and all participants provided written informed consent prior to participating in the study.

### 2.2. Determination of ABI

All measurements were obtained after the patients had rested for ten min in the supine decubitus position in a room with a comfortable temperature (24 ± 1°C), without smoking, heavy exercise, and drinking alcohol or caffeinated beverages for at least 2 h before the examination. The ABI was determined by the automatic method using a validated oscillometric device (OMRON BP-203RPEIII) that allows simultaneous arm-leg BP measurements and using a validated and calibrated sphygmomanometer and a two-way Doppler with an 8 MHz probe (Bidop Es-100V3. HADECO) by the same specially trained nurse with 10 years of experience in ABI measurement. Doppler-ABI measurement was invariably performed first because of the higher degree of subjectivity. For oscillometric ABI, they used appropriate cuff sizes on the arms and ankles, thus avoiding a potential bias by variations of blood pressure. Ankle pressures were measured over the dorsalis and posterior tibial arteries. Limbs were measured twice at the same time and the time interval was 10 seconds. ABI was calculated by dividing the highest value obtained at each ankle by the highest of the arm values. The ABI of both the left and right legs was recorded, and for the definition of PAD, the lower value between the two was considered [22].

### 2.3. Statistical Analysis

The results were analyzed by diagnostic test analysis method. Continuous variables are summarized as mean ± standard deviation (SD) when normally distributed and as median (interquartile range) when asymmetrically distributed and categorical variables as percentage. ABI measurements were compared using a paired Student's *t*-test. The intermethod concordance between both techniques was assessed by kappa coefficient and the Bland-Altman method was determined to analyze the agreement. Diagnostic accuracy was assessed via sensitivity, specificity, accuracy, positive likelihood ratio (+LR), negative likelihood ratio (−LR), with ABI readings dichotomized (ABI ≤ 0.9), and receiver operating characteristic (ROC) curve analysis using both univariable and multivariable logistic regressions. Conventionally, Cohen's kappa statistic below 0.2 is considered poor agreement, 0.21–0.4 fair, 0.41–0.6 moderate, 0.61–0.8 strong, and over 0.8 near complete agreement [23]. *p* < 0.05 was accepted as indicating statistical significance. The statistical analysis was carried out using the SPSS statistics package, version 16.0 (SPSS Inc., Chicago, IL, USA) and MedCalc 15.8 software.

## 3. Results

### 3.1. Patient Characteristics

Patient characteristics are shown in Table 1.

### 3.2. Comparison of the Two Methods

**Figure 2:**
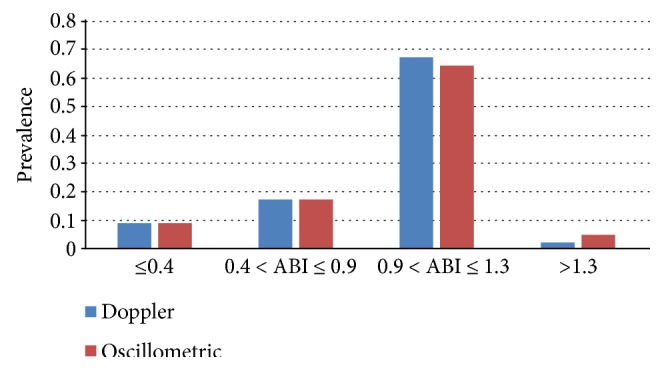
Prevalence across the categories of Doppler and oscillometric ABI values.

ABI values measured by eco-Doppler showed 1.003 ± 0.286 (0.210–1.390) on the right limb and 0.990 ± 0.287 (0.000–1.000) on the left limb, while the automated oscillometric measurement showed 1.002 ± 0.332 (0.000–1.900) on the right and 0.993 ± 0.319 (0.000–1.390) on the left. The difference of the oscillometric ABIs and the Doppler ABIs was not significant on the right (95%CI = −0.0203–0.0185, *p* = 0.930) and left legs (95% CI = −0.0146–0.0209, *p* = 0.727). Pathological ABI by eco-Doppler was detected in 67 (29.13%) subjects, including 43 subjects with abnormal ABI at two limbs and 24 subjects at one limb and in 66 (28.70%) subjects using the automated oscillometric measurement including 43 subjects at two limbs and 23 subjects at one limb. The prevalence across the categories of Doppler and oscillometric ABI values are shown in Figure 2; no significant differences were observed for ABI ≤ 0.4, 0.4 < ABI ≤ 0.9, 0.9 < ABI ≤ 1.3, and ABI > 1.3 (*x*^2^ = 2.703, *p* = 0.259). Compared to ABI obtained by eco-Doppler, the automated oscillometric measurement produced a false positive result in 6 (8.96%) patients and a false negative result in 6 (3.82%) patients.

### 3.3. The Agreements between Doppler and Automatic Methods

**Figure 3:**
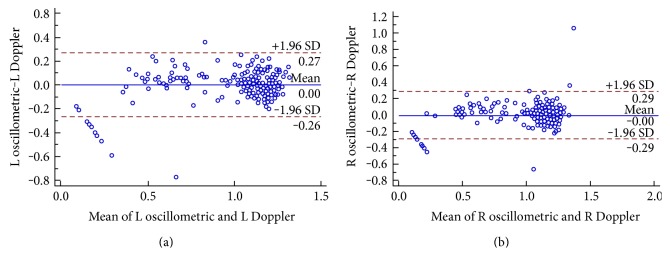
The Bland-Altman plots for the ABI by oscillometric and Doppler devices.

The Bland-Altman plots of the difference comparison assessing the agreement of the two methods for all 230 patients are shown in Figure 3; the paired mean (95% confidence interval (CI)) difference between two measuring devices according the Altman-Bland method was −0.0009 (95% CI = −0.2993 to 0.2976, *p* < 0.0005) in the right limbs and 0.0031(95% CI = −0.2692 to 0.2755, *p* < 0.0001) in the left limbs. The value-to-value comparison showed good agreement between the two methods.

### 3.4. Sensitivity and Specificity

**Figure 4:**
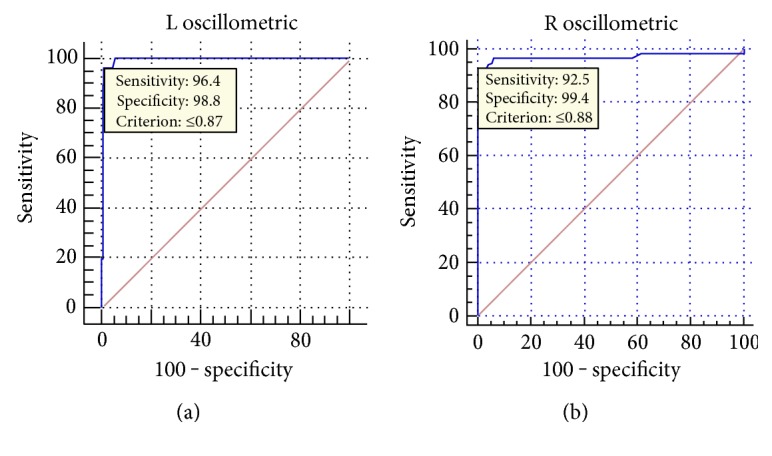
The area under the receiver operating characteristic curve for ABI measured by oscillometric analysis compared with that measured by Doppler analysis.

Regarding the eco-Doppler measurement as the gold standard (defined ABI ≤ 0.9 as PAD), there were 173 true negatives, 50 true positives, 4 false negatives, and 3 false positives in the right legs, and 13 patients with an automatic index that was not measurable were classified correctly as true positives. There were 171 true negatives, 53 true positives, 2 false negatives, and 3 false positives in the left legs, and there were 11 patients with an automatic index that was not measurable. While in the analysis in terms of patients rather than limbs in the 230 patients with determination of ABI by both methods, there were 157 true negatives, 61 true positives, 6 false negatives, and 6 false positives, and there were 16 patients with an automatic index that was not measurable. Sensitivity, specificity, positive likelihood ratio, negative likelihood ratio, accuracy, and kappa values of the oscillometric method are shown in Table 2. The area under the receiver operating characteristic (ROC) curve was 0.993 (95% CI = 0.972 to 1.000) on the left limbs and 0.967 (95% CI = 0.935 to 0.986) on the right limbs (Figure 4()); in the combined analysis, the area under the ROC was 0.981 (95% CI = 0.964 to 0.991).

### 3.5. Comparison of Time Consumption

The mean time consumption for measurements with the automatic device and the handheld Doppler device was 8.60 ± 1.38 (ranges: 7.00–14.00) and 16.98 ± 3.20 (ranges: 10.00–30.00) minutes per patient, respectively. Compared to the handheld Doppler, the process of performing the automated oscillometric device consumed significantly less time (*p* < 0.001).

## 4. Discussion

Our previous studies indicated that PAD existed in “three high” (high prevalence, high morbidity, and high mortality) and “three low” (low diagnosis, low treatment, and low awareness) conditions [24]. PAD was a risk factor of diabetic foot and one of the independent risk factors of diabetic foot amputation [25, 26]. Thus, early detection of PAD is necessary to prevent diabetic foot and amputation. The validity and reliability of automated oscillometric ABI values for diagnosis of PAD is controversial [14–21, 23]. Although the Doppler-derived ABI was the gold standard to ABI, oscillometric-ABI measurement is simple and convenient with low cost for PAD screening [21, 27]. However, few studies have validated the concordance between oscillometric ABI and Doppler ABI in China.

In this study, we found that the automated oscillometric-ABI-measuring results was highly consistent with those by eco-Doppler methods and the former is less time consuming. The previous studies showed the correlation between the two methods ranging from 0.53 to 0.86 [19–23]. This study showed that the kappa value was 0.869 and 0.919 for the right and left legs, respectively. Using the Doppler-derived ABI as the gold standard and that the cutoff value was 0.9, the sensitivity and specificity of the oscillometric method are 94.50% and 98.29%, respectively, with the receiver operating characteristic (ROC) curve being 0.992. Most recently, Herráiz-Adillo et al. [28] published a similar study (43% diabetics, *n* = 151 legs), which showed a sensitivity of 66.7%, a specificity of 96.8%, and an accuracy of 91.39%, with a kappa coefficient of 0.684 and AUC = 0.944 (95% CI = 0.905 to 0.983). Furthermore, when they were considering calcified legs as PAD equivalents (*n* = 180 legs), the sensitivity, specificity, and accuracy were 78.2%, 96%, and 90.56%, respectively, with a kappa coefficient of 0.645 and AUC = 0.914 (95% CI = 0.872 to 0.955). The differences between the study of Herráiz-Adillo et al. and our study maybe due to the different selected subjects and the detection equipment (OMRON M3 versus OMRON BP-203RPEIII). Especially, high sensitivity (90%) and specificity (98%) of oscillometric test for stenosis of ≥50% in the arteries of the lower limbs were found in this and other studies [29]. However, some studies [29, 30] showed that oscillometry overestimated ankle pressure and the ABI result was unreliable. Actually, ABI values were comparatively lower while the ABI values ≤ 0.4 compared with eco-Doppler. There are 23 cases which ABI < 0.4, while 16 of them cannot be detected by oscillometric measurement. Therefore, if the oscillometric device cannot detect specific ABI data, it indicated that the lower limb artery lesion was severe with ABI < 0.4. Further examinations such as CTA, MRA, and angiography were needed to evaluate and treatment of PAD must be initiated.

The time needed for Doppler ABI was longer than that for oscillometric-ABI methods (16.98 ± 3.20 (10.00–30.00) min versus 8.60 ± 1.38 (7.00–14.00) min). It may be due to the necessity of additional steps with Doppler, such as pulse palpation, the application of gel, signal viewing, and operational levels. The different results between the two methods were acceptable. The eco-Doppler method was affected by the operator and the intrinsic bias existed during measurement. And the automated oscillometric-ABI value was not influenced by the operator and the results were more reliable.

Most published researches were based on nondiabetic population and suggested that an automated oscillometric ABI measurement is a reliable and practical alternative to the conventional Doppler measurement for the detection of PAD. Our objectives of the study were involved diabetic patients to validate. Our study was a single-center study and a sample size was not enough; further studies need to be performed.

In conclusion, our finding suggested that ABI values measured by automated oscillometric method were highly consistent with those by eco-Doppler method. The former was convenient and less time consuming, which can be widely used in the primary care center without special training.

## Figures and Tables

**Table 1:** Patient demographics and clinical characteristics.

Variable	Value
Male (%)	126/230 (54.78%)
Age (years)	61.28 ± 14.50
Duration of diabetes (years)	8.97 ± 6.77
BMI (kg/m^2^)	23.50 ± 3.97
HbA1c (%)	8.83 ± 2.54
FBG (mol/L)	9.35 ± 4.02
CHOL (mol/L)	4.41 ± 1.30
TG (mol/L)	1.88 ± 1.70
HDL-C (mol/L)	1.20 ± 0.37
LDL-C (mol/L)	2.54 ± 1.08
Creatinine (mol/L)	88.31 ± 50.12
Intermittent claudication	45/230 (19.57%)
Rest pain	32/230 (13.91%)
Coronary heart disease	98/230 (42.61%)
Cerebrovascular disease	50/230 (21.74%)
Retinopathy	103/230 (44.78%)
Neuropathy	158/230 (68.70%)
Hypertension	154/230 (66.96%)
Hypercholesterolemia	16/320 (5.00%)
Hypertriglyceridemia	52/320 (22.61%)
Peripheral arterial disease	56/230 (24.35%)
Diabetic foot	82/230 (35.65%)
Wagner grade 1	3/82 (3.66%)
Wagner grade 2	12/82 (14.63%)
Wagner grade 3	40/82 (48.78%)
Wagner grade 4	25/82 (30.49%)
Wagner grade 5	2/82 (2.44%)

BMI: body mass index; FBG: fasting blood glucose; CHOL: cholesterol; TG: triglyceride; HDL-C: high density lipoprotein cholesterol; LDL-C: low density lipoprotein cholesterol.

**Table 2:** Accuracy of the oscillometric method compared with that of the gold standard.

	Accuracy	Sensitivity	Specificity	+LR	−LR	*p*	Kappa
Right limb	95.22%	94.34%	95.48%	20.87	0.059	0.000	0.869
Left limb	96.94%	96.43%	97.11%	33.36	0.036	0.000	0.919
Combined analysis	97.37%	94.50%	98.29%	55.12	0.056	0.001	0.928
